# Efficacy of alogliptin in type 2 diabetes treatment: a meta-analysis of randomized double-blind controlled studies

**DOI:** 10.1186/1472-6823-13-9

**Published:** 2013-03-01

**Authors:** Asres Berhan, Yifru Berhan

**Affiliations:** 1 Hawassa University College of Medicine and Health Sciences, Hawassa University, P. O. Box: 1560, Hawassa, Ethiopia

**Keywords:** Alogliptin, Body weight, DPP-4 inhibitors, FPG, HbA1c, Meta-analysis, Type 2 diabetes

## Abstract

**Background:**

Alogliptin is a new dipeptidyl peptidase (DPP-4) inhibitor, which is under investigation for treatment of type 2 diabetes either alone or in combination with other antidiabetic drugs. The aim of this meta-analysis was to assess the efficacy and tolerability of alogliptin in patients with type 2 diabetes.

**Methods:**

Computer based search was performed in MEDLINE, Cochrane library, and HINARI (Health InterNetwork Access to Research Initiative) databases. Meta-analysis was carried out by incorporating double-blind randomized controlled studies done on the efficacy of alogliptin in patients with type 2 diabetes. The efficacy and tolerability of alogliptin was determined by standardized mean differences (SMDs) and Mantel-Haenszel odds ratio. Heterogeneity was assessed by the chi-squared test (Cochran Q test) and I^2^ statistics.

**Results:**

The pooled SMDs demonstrated a significant reduction in HbA1c in patients treated with alogliptin 12.5 mg (SMD = −0.81; 95% CI, -1.11 to −0.51) or alogliptin 25 mg (SMD= −0.98; 95%CI= −1.30 to −0.66) as compared with controls. The SMD for reduction in fasting plasma glucose level (FPG) from baseline was also statistically significant among alogliptin treated patients. However, the effect of alogliptin on body weight change was inconclusive. The proportion of patients who discontinued alogliptin due to adverse events was not different from controls. Similarly, the meta-analyses of specific adverse events did not demonstrate statistically significant differences.

**Conclusions:**

Alogliptin alone or in combination with other antidiabetic drug has shown a significant reduction in HbA1c and FPG level in patients with type 2 diabetes. However, its consistent efficacy for longer duration of therapy needs further investigation.

## Background

Type 2 diabetes is characterized by insulin resistance accompanied by progressive loss of pancreatic β-cells function [1,2]. Additionally, patients with type 2 diabetes secret smaller amount of glucagon-like peptide–1 (GLP-1) and have a decreased insulinotropic effect of glucose dependent insulinotropic polypeptide (GIP) [3]. The incretin hormones (GLP-1 and GIP) are produced in the small intestine in response to food intake, and then stimulate glucose dependent insulin secretion from pancreatic β-cells [4]. The stimulatory effect of GLP-1 is short lasting due to a rapid inactivation by the widely distributed DPP-4. The search for drugs that are able to mimic incretin hormones or prolong the half-life of incretins has led to the discovery of incretin hormone mimetics (exenatide and liraglutide) and DPP-4 inhibitors (sitagliptin, vildagliptin, saxagliptin, linagliptin and alogliptin).

Among the DPP-4 inhibitors, alogliptin is still under investigation for treatment of type 2 diabetes as monotherapy or in combination with other antidiabetic drugs. Pharmacokinetic studies showed that alogliptin is absorbed rapidly in the small intestine, and primarily excreted via the renal system in unchanged form [5,6]. This drug has got approval for treatment of type 2 diabetes in Japan [7,8] and not yet approved by the Food and Drug Administration (FDA) of America in till this manuscript is written [9].

The prevailing approach to the treatment of type 2 diabetes is starting with single oral antidiabetic drug followed by dose escalation and then combination therapy [10]. However, there is a growing consensus on the earlier initiation of insulin therapy and the use of combination oral agents including incretin mimetics [10].

A recent systematic review and meta-analysis that assessed the long term safety of DPP-4 inhibitors relative to placebo has reported an insignificant adverse events and risk of infections [11]. However, the aim of this meta-analysis was to assess the efficacy and tolerability of alogliptin (25 mg and 12.5 mg), which is not yet approved in many countries as monotherapy and/or add-on therapy in patients with type 2 diabetes. The primary outcome indicators of alogliptin efficacy were change in percentage of HbA1c and FPG level from the baseline.

## Methods

### Search strategy

Computer based search for literature on alogliptin was performed by AB in MEDLINE, Cochrane library, and HINARI databases. Via HINARI, literature search were also conducted on publishers’ websites (Elsevier Science-Science Direct, Nature Publishing Group, Oxford University Press, PsycARTICLES, Science, Wiley-Blackwell and Springer Link). The search was further strengthened by searching relevant literature from the reference lists of retrieved articles. The search terms include: alogliptin or NESINA® or SYR-322, type 2 diabetes mellitus, dipeptidyl peptidase-4 (DPP-4) inhibitors, hemoglobin A1C, FPG and body weight. During searching, the term alogliptin was used alone and in an alternate combination with other search terms with the help of Boolean logic (and/or).

### Study selection

The inclusion criteria for this meta-analysis were: 1) double-blind randomized controlled studies that weighted the efficacy and tolerability of alogliptin against placebo or other antidiabetic drugs in patients with type 2 diabetes; 2) studies that were published in English and have a duration of therapy not less than 12 weeks; and 3) studies which recruited patients with baseline glycosylated hemoglobin level (HbA1c) ≥ 7% and studies that provide information on change in percentage of HbA1c for every treatment group (alogliptin 12.5 mg, alogliptin 25 mg and placebo or other drug). The study selection was independently conducted by both authors. When there were discrepancies, it was resolved by discussion and by reviewing the studies in detail.

### Data extraction

After developing a common data extraction template, the following information were abstracted from the selected studies by both authors separately with standard Excel spreadsheet: name of authors, year of publication, study design, study location, drugs before and after recruitment, duration of therapy, sample size, least squared (LS) mean and standard deviation (SD) or standard error (SE) of changes (in hemoglobinA1c, FPG, body weight and serum lipids), number of patients achieving HbA1c ≤ 7%, number of patients with reduction of HbA1c by ≥ 1.0%, number of discontinued patients due to adverse event, and number of patients with a specific adverse events.

### Operational definitions

In the selected studies, controls could receive placebo or other antidiabetic drug or other antidiabetic drug plus placebo. In this meta-analysis, the term alogliptin alone is to mean that patients were given either only alogliptin 25 mg or 12.5 mg. Similarly, alogliptin add-on is to mean that patients received either alogliptin 12.5 mg or 25 mg plus other antidiabetic drugs. Antidiabetic naïve is to mean patients who were not on antidiabetic drug; while antidiabetic drug experienced patients were on antidiabetic therapy before the start of the studies.

### Data synthesis & statistical analysis

Before the actual meta-analysis was conducted, some mathematical transformations and unit conversions were done. In case of continuous variables (change in HbA1c, FPG, body weight and serum lipids), where SE was reported instead of SD, we changed it to SD by multiplying the SE by the square root of sample size (SD = SE*√N). In studies where the change in FPG was reported as mg/dl, it was converted to mmol/l by using an online converter [12]. In one study, we have extrapolated values for reduction of HbA1c by ≥ 1.0% from a bar graph [13].

The effectiveness and tolerability of the two doses of alogliptin (12.5 mg and 25 mg) alone and as an added-on with other antidiabetic drug as compared to placebo or other antidiabetic drug were determined using the random effects model. SMDs and Mantel-Haenszel (M-H) odds ratios were determined. SMD and 95% confidence intervals (95% CI) for the mean change in HbA1c, FPG, body weight and serum lipids from baseline were computed using the inverse variance method. The odds ratios and the 95% confidence intervals for achieving HbA1c ≤ 7%, reduction HbA1c by ≥ 1%, treatment discontinuations due to adverse events and experiencing adverse events (hypertension, hypoglycemia, skin or subcutaneous adverse events etc.) were computed with Mantel-Haenszel method.

To assess the heterogeneity among the studies, chi-squared test (Cochran Q test) and I^2^ statistics were used. An I^2^ value of ≥ 50% was considered as statistically significant. Subgroup analysis based on the use of alogliptin as monotherapy or add-on therapy (alogliptin alone vs alogliptin with other antidiabetic drug), patients’ antidiabetic drug exposure history (antidiabetic drug experienced vs antidiabetic naïve) and the sites of the studies (studies in a single country at multiple sites vs multiple country studies) were planned and conducted. On the other hand, meta-regression was limited to one covariate (duration of therapy) to avoid false-positive findings. Sensitivity analysis was also conducted to see the stability of the pooled values and the change in I^2^ when any of the study was withdrawn from the analysis.

Risk of bias of individual studies was assessed with the Cochrane risk of bias tool. The predefined key domains include: random sequence generation, allocation concealment, blinding of participants and personnel, blinding of outcome assessment, incomplete outcome data, selective reporting and other bias. A bias that is unlikely to affect the result was considered as “low risk of bias”, while a bias that raises doubt about the results was considered as “unclear risk of bias” and bias that seriously affect the results was considered as “high risk of bias”. To evaluate publication/disclosure bias, we have used funnel plots. Nevertheless, the tests for funnel plot asymmetry were not done as recommended in meta-analyses of randomized controlled trials with fewer than ten studies [14]. We reported the meta-analysis by following the PRISMA checklist [15]. The analyses were conducted with Review Manager (RevMan) Version 5.1 software [16] and Comprehensive Meta-Analysis Software [17].

## Results

**Figure 1:**
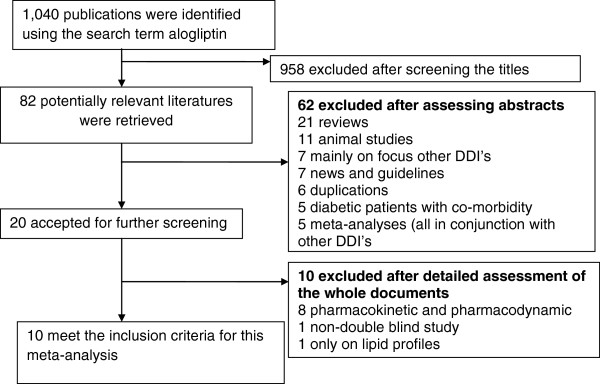
Flow diagram to show studies selection process, 2012.

From the retrieved 82 publications on alogliptin, only ten published articles met the inclusion criteria (Figure 1). Seven of the studies were done in multiple-countries [13,18-23]; whereas three of the studies were done in a single country at multiple sites (all in Japan) [24-26] (Table 1). Six of the studies compared the effectiveness of alogliptin as add-on to other antidiabetic drug(s) against antidiabetic drug with placebo or without placebo [18-20,23,24,26] and the remaining four compared alogliptin alone with placebo [13,21,22,25]. The selected ten studies have included 4,339 patients with type 2 diabetes; 1,707 received alogliptin 25 mg alone or as add-on; 1,311 received alogliptin 12.5 mg alone or as add-on and the remaining 1,321 received placebo or other antidiabetic drug(s) with or without placebo. The risk of bias assessment in the selected studies demonstrated that there were no biases in randomization, blinding and selective reporting. However, other sources of bias cannot be ruled out.

**Table 1:** Characteristic of studies included in this Meta-analysis, 2012

**Authors**	**Year**	**Country**	**Duration**	**Medication before recruitment**	**Group1: alogliptin 12.5 mg +**	**Group2: alogliptin 25 mg +**	**Group 3 (controls)**
Rosenstock et al., Study 1 [13]	2009	Multiple	26wks	Insulin ± metformine	Alone	Alone	Placebo
Bosi et al., [17]	2011	Multiple	52 wks	Metformine + pioglitazone	--	Metformine + pioglitazone	Metformine + pioglitazone
Nauck et al., [18]	2009	Multiple	26wks	Metformin	Metformin	Metformine	Metformine + placebo
Kaku et al., [23]	2011	Japan	12wks	Pioglitazone + diet + exercise	Pioglitazone	Pioglitazone	Piogitazone + placebo
Seino et al., Study 1 [24]	2011	Japan	12wks	Diet +exercise	Alone	Alone	placebo
Pratley et al., Study 1 [19]	2009	Multiple	26wks	TZD ± metformin or sulfonylureas	Pioglitazone	Pioglitazone	Pioglitazone
Pratley et al., Study2 [20]	2009	Multiple	26wks	Sulfonylureas	Alone	Alone	Placebo
Defronzo et al., [21]	2008	Multiple	26wks	Diet +exercise	Alone	Alone	Placebo
Rosenstock et al., Study 2 [22]	2010	Multiple	26wks	Diet +exercise	Pioglitazone	Pioglitazone	Pioglitazone
Seino et al., Study 2 [25]	2011	Japan	12wks	α-glucosidase inhibitor +diet	Voglibose	Voglibose	Voglibose

**Figure 2:**
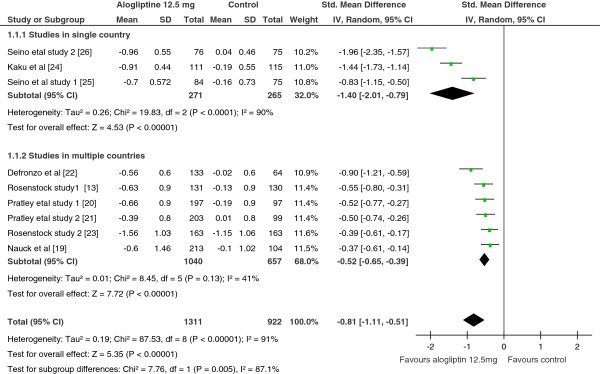
Standardize mean difference of the change in HbA1c from subgroup analysis by study location, Alogliptin 12.5 mg vs Control, 2012.

**Figure 3:**
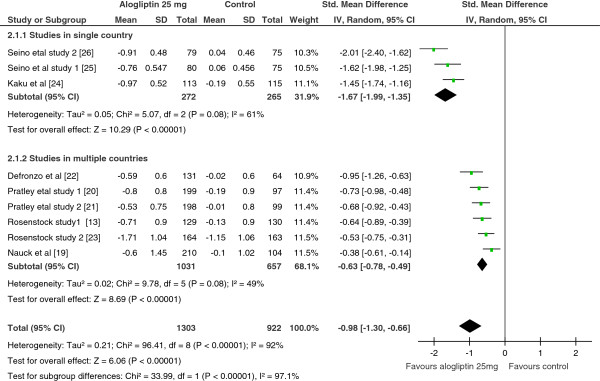
Standardize mean difference of the change in HbA1c from subgroup analysis by study location, Alogliptin 25 mg vs Control, 2012.

As shown in Figure 2 and 3, the pooled SMDs for alogliptin 12.5 mg treated vs controls, and alogliptin 25 mg treated vs controls demonstrated a significant reduction in HbA1c in patients treated with either dose of alogliptin (SMD = −0.81; 95% CI, -1.11 to −0.51; SMD= −0.98; 95% CI= −1.30 to −0.66 for alogliptin 12.5 mg vs controls and alogliptin 25 mg vs controls, respectively). However, heterogeneity test and sensitivity analysis showed the existence of a significant heterogeneity among the included studies and no improvement of heterogeneity with withdrawal of any of the study from the analysis, respectively. When any of the study withdrawn from the analysis, the pooled SMD swings between −0.87 and −0.68 for alogliptin 12.5 mg, and between −1.06 and −0.86 for alogliptin 25 mg.

From the pre-specified subgroup analyses, the use of both doses of alogliptin as add-on or alone (alogliptin alone vs alogliptin plus other antidiabetic drug) and patients difference in antidiabetic drug treatment experience (antidiabetic drug naive vs antidiabetic drug experienced) did not show a statistically significant variation. In other words, the treatment outcomes were not significantly different when alogliptin was used as monotherapy or as add-on therapy. Similarly, the treatment outcome was not dependent on patients’ antidiabetic drug experience. However, the subgroup analysis showed a significant reduction in HbA1c in single country studies than studies in multiple countries.

Furthermore, the meta-regression on the influence of duration of alogliptin therapy (both 25 mg and 12.5 mg) on the reduction of HbA1c revealed that as duration of therapy gets longer, the effect on HbA1c reduction become minimal, which was a highly statistically significant (slope = 0.06, 95% CI = 0.033 to 0.09 and P-value < 0.0001 for alogliptin 12.5 mg; slope = 0.07, 95% CI = 0.05 to 0.095 and P-value < 0.0001 for alogliptin 25 mg).

In comparison to controls, the proportion of patients achieving HbA1c ≤ 7% was significantly higher in patients treated with either doses of alogliptin (OR = 2.4; 95% CI, 1.66 to 3.57 for 12.5 mg; OR = 2.4; 95% CI, 1.89 to 3.10 for 25 mg ). Similarly, when both doses compared separately with controls, the proportions of patients with ≥1% reduction of HbA1c from baseline were also significantly higher with alogliptin treated than controls (OR = 2.6; 95% CI, 1.83 to 3.56 for 12.5 mg; OR = 3.3; 95% CI, 2.54 to 4.28 for 25 mg).

**Figure 4:**
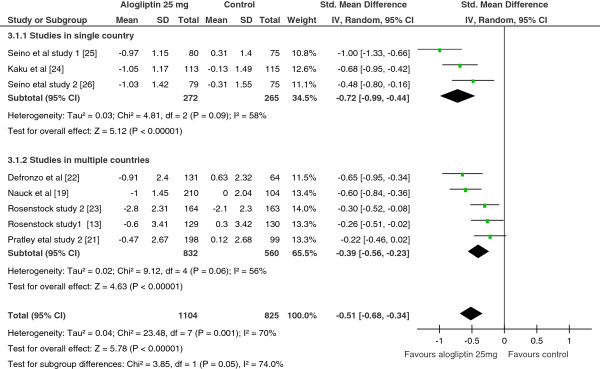
Standardize mean difference of the change in FPG level from a subgroup analysis based on study location, Alogliptin 25 mg vs Control, 2012.

The pooled SMD in alogliptin 12.5 mg treated vs controls and alogliptin 25 mg treated vs controls also showed a statistically significant reduction in FPG level among patients treated with either dose of alogliptin (SMD = −0.43, 95% CI = −0.6 to −0.26; SMD = −0.51, 95% CI = −0.68 to −0.34 for alogliptin 12.5 mg and alogliptin 25 mg, respectively). The heterogeneity test still showed a significant variation among the included studies (Figure 4). Sensitivity analysis showed that the SMD changed by about 0.05 at maximum with the withdrawal of any of the included study but no improvement of heterogeneity.

**Figure 5:**
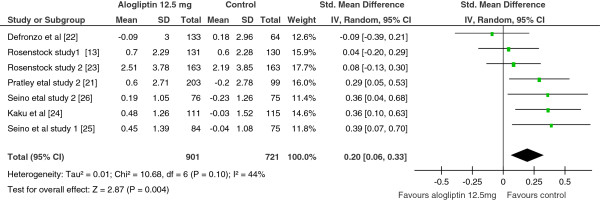
Standardize mean difference of change in body weight, Alogliptin 12.5 mg vs Control, 2012.

The findings from the analysis of change in body weight in alogliptin 25 mg vs controls illustrated no difference. However, with moderate heterogeneity, the analysis of alogliptin 12.5 mg vs control showed a significant weight increment among the alogliptin 12.5 mg treated patients (SMD = 0.20; 95% CI, 0.06 to 0.33) (Figure 5).

**Figure 6:**
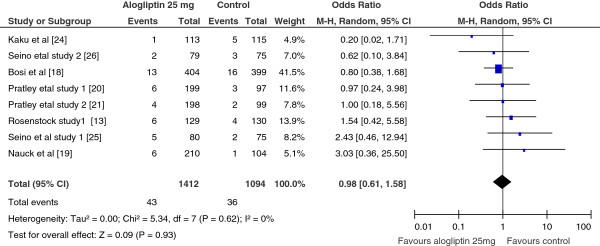
Mantel-Haenszel odds ratio of patients who discontinued the treatment due to adverse events, Alogliptin 25 mg vs Control, 2012.

Comparisons based on the number of patients who discontinued treatment due to adverse events in alogliptin 12.5 mg vs controls and alogliptin 25 mg vs controls were not significantly different. In other words, the number of patients who discontinued due to adverse events in the alogliptin treated group were not different from placebo or other antidiabetic drug treated (OR = 0.83; 95% CI, 0.61 to 1.58 for alogliptin 12.5 mg; and OR=0.98; 95% CI, 0.44 to 1.58 for alogliptin 25 mg (Figure 6).

Similarly, as shown in Table 2, the proportion of patients who experienced adverse events or infections (skin or subcutaneous adverse events, headache, arthralgia, nasopharyngitis, diarrhoea, peripheral oedema, gastrointestinal adverse events, urinary tract infection, upper respiratory tract infection, infection and infestation, bronchitis, influenza, hypoglycaemia, and hypertension) were not significantly different from controls. Furthermore, the effect of either dose of alogliptin (12.5 mg and 25 mg) on serum lev of total cholesterol, LDL-cholesterol, HDL-cholesterol and triglycerides was not also significantly different from controls (Table 3).

**Table 2:** Mantel-Haenszel odds ratios of different adverse events (Alogliptin 25 mg vs control; Alogliptin 12.5 mg vs control), 2012

**Alogliptin 25 mg treated vs control**			
Adverse events	Over all odds Ratios (95% CI)	I^2^	Number of included studies
Any skin or subcutaneous adverse events	0.91 [0.60, 1.40]	0%	3 [13,20,21]
Headache	1.07 [0.64, 1.78]	0%	4 [13,18-20]
Arthralgia	0.79 [0.37, 1.72]	24%	3 [13,18,19]
Nasopharyngitis	1.34 [0.92, 1.95]	0%	6 [13,18-21,24]
Diarrhoea	1.36 [0.34, 5.49]	74%	4 [13,18,19,21]
Peripheral oedema	0.98 [0.60, 1.61]	0%	4 [13,18,20,24]
Gastrointestinal adverse events	1.04 [0.74, 1.46]	0%	4 [13,19-21]
Urinary tract infection	0.98 [0.54, 1.77]	45%	4 [13,18,19,21]
Upper respiratory tract infection	0.77 [0.32, 1.86]	67%	4 [18-21]
Infection or infestation	0.87 [0.67, 1.13]	0%	4 [13,19-21]
Bronchitis	0.72 [0.24, 2.14]	58%	4 [18-21]
Influenza	0.83 [0.49, 1.38]	0%	3 [18,20,21]
Hypoglycaemia	0.97 [0.23, 4.01]	75%	3 [18,19,21]
Hypertension	1.09 [0.57, 2.08]	22%	3 [18,19,21]
**Alogliptin 12.5 mg treated vs control**			
Any skin or subcutaneous adverse events	0.86 [0.56, 1.33]	0%	3 [13,20,21]
Headache	1.23 [0.59, 2.55]	0%	3 [13,19,20]
Arthralgia	1.08 [0.14, 8.57]	79%	2 [13,19]
Nasopharyngitis	0.86 [0.50, 1.46]	0%	5 [13,19-21,24]
Diarrhoea	1.15 [0.19, 6.80]	50%	3 [13,19,21]
Peripheral oedema	0.80 [0.37, 1.72]	0%	3 [13,19,24]
Gastrointestinal adverse events	0.88 [0.62, 1.24]	0%	4 [13,19-21]
Urinary tract infection	1.17 [0.62, 2.23]	0%	3 [13,19,21]
Upper respiratory tract infection	0.66 [0.34, 1.27]	5%	3 [19-21]
Infectionn or infestation	1.01 [0.78, 1.31]	0%	4 [13,19-21]
Bronchitis	0.72 [0.24, 2.16]	38%	3 [19-21]
Influenza	0.42 [0.15, 1.17]	0%	2 [20,21]
Hypoglycaemia	0.87 [0.20, 3.69]	59%	2 [19,21]
Hypertension	0.76 [0.17, 3.38]	52%	2 [19,21]

Note. Numbers in parenthesis of the last column indicate the included studies references number.

**Table 3:** Standardize mean differences of the change in serum lipids level from baseline (Alogloptin 25 mg vs control; Alogloptin 12.5 mg vs control), 2012

**Alogliptin 25 mg treated vs control**			
Lipid profile	Std. Mean Difference (95% CI)	I^2^	Number of included studies
Change in total cholesterol	−0.16 [−0.46, 0.15]	76%	4 [22,24-26]
Change in LDL-cholesterol	−0.05 [−0.27, 0.16]	53%	4 [22,24-26]
Change in HDL-cholesterol	−0.17 [−0.32, -0.02]	0%	4 [22,24-26]
Change in triglycerides	−0.14 [−0.33, 0.05]	39%	4 [22,24-26]
**Alogliptin 12.5 mg treated vs control**			
Change in total cholestrol	−0.13 [−0.37, 0.10]	60%	4 [22,24-26]
Change in LDL-cholestrol	−0.10 [−0.25, 0.05]	0%	4 [22,24-26]
Change in HDL-cholesterol	−0.13 [−0.28, 0.02]	0%	4 [22,24-26]
Change in triglycerides	−0.06 [−0.29, 0.17]	58%	4 [22,24-26]

Note. Numbers in parenthesis of the last column indicate the included studies references number.

## Discussion

In agreement with a previous meta-analysis, that was conducted on DPP-4 inhibitors including four studies on alogliptin [27], this meta-analysis has demonstrated a significant reduction of HbA1c and FPG in patients treated with either alogliptin 12.5 mg or alogliptin 25 mg. Moreover, addition of either dose of alogliptin to a previously prescribed antidiabetic drug(s), in patients with inadequately controlled type 2 diabetes, has shown a statistically significant reduction in HbA1c and FPG - better than the previously prescribed antidiabetic drug(s) alone. Since the FPG reflects the hepatic glucose production, which depends on insulin secretory capacity of the pancreas [28], while the HbA1c provides information about the degree of long-term glycemic control [29], the finding of reduction in both markers with alogliptin probably indicates its short and long term efficacy.

Although heterogeneity testing showed a statistically significant dissimilarity in the results of the included studies, sensitivity analysis has shown the stability of the overall odds ratios with the withdrawal of any of the study from the analysis without a significant improvement of the heterogeneity. Thus, the credibility of the results of this meta-analysis did not seem compromised. This is because; when the number of included studies is small and heterogeneity is large, the robustness of the results is best assessed with a sensitivity analysis [30].

Meta-regression showed a negative relation between the duration of alogliptin therapy and its efficacy (HbA1c reduction become minimal). However, this does not necessarily mean that alogliptin is ineffective after 12 weeks of therapy. Rather, its long term efficacy needs further investigation. From the subgroup analysis, the effect of alogliptin add-on therapy (alogliptin plus other antidiabetic drug) in lowering HbA1c does not appear superior to alogliptin monotherapy. However, this finding should be interpreted very cautiously. This is because; two of the studies in the alogliptin monotherapy subgroup recruited patients with type 2 diabetes who were naïve to antidiabetic drugs [22,25]. As a result, patients with type 2 diabetes who were not antidiabetic drug experienced could probably respond better to alogliptin therapy. Additionally, prior study on a combination of other DPP-4 inhibitor (sitagliptin) with metformin has shown better glycemic control than DPP-4 inhibitor monotherapy [31].

On the other hand, the achievement of HbA1c ≤ 7% and HbA1c reduction by ≥ 1% brought further evidence in support of alogliptin efficacy in lowering elevated HbA1c. However, the reason for a significantly higher reduction of HbA1c in studies which were conducted in one country (all in Japan) than studies which were conducted in multiple countries is not exactly known. The possible explanation could be: 1) the shortness of the duration of therapies; i.e. unlike the other multi-country studies included in this meta-analysis (with a minimum duration of therapy 26 weeks), Japanese studies that were included in this meta-analysis have duration of therapy only 12 weeks. In support of this assumption, the meta-regression has shown more reduction in HbA1c in the 12 weeks therapy than the 26 weeks therapy. 2) The sample sizes of the studies which were conducted in Japan were relatively smaller than other studies which were conducted in multiple countries. It is known that small sample size studies have low power to establish the true research finding [32]. 3) There may be difference in ethnic and cultural background, which may have an influence on the treatment outcomes. Accordingly, Japanese patients with type2 diabetes could respond better to alogliptin than others. In support of our opinion, previous studies on other drugs have shown that there was difference between Japanese and Caucasians in pharmacokinetic and pharmacogenetic parameters for unknown reason [33,34].

This meta-analysis also showed a significant reduction in FPG level among alogliptin treated groups, that is a desirable effect of any antidiabetic drug. From the theorized causes of diabetes mellitus complications, the widely accepted is persistent hyperglycemia that leads to spontaneous glycosylation of amino acids, lipids and nucleic acid [35]. As a result, the major goals in the therapy of diabetes mellitus is to keep the plasma glucose level below 110 mg/dl and maintain HbA1c < 7% [36,37]. This meta-analysis demonstrated the efficacy of alogliptin even in poorly controlled diabetes with other antidiabetic drug(s). In other words, four of the included studies in FPG and five studies in the HbA1c analyses were conducted in patients who were on other antidiabetic drugs but with poorly controlled diabetes.

Patients with type 2 diabetes are usually overweight or obese [38]. Unluckily, some other antidiabetic drugs like sulfonylureas, meglitinides, thiazolidinediones and insulin are known to be associated with weight gain, and may aggravate the already gained weight [39,40]. However, the effect of alogliptin 25 mg on body weight was not different from the controls (placebo or other antidiabetic drug treated). But, the pooled analysis on body weight change among the alogliptin 12.5 mg treated group demonstrated a significant weight gain. The result should be considered with great caution and needs further investigation. This is because, previous meta-analysis on the effect of DPP-4 inhibitors on body weight showed that DPP-4 inhibitors were weight neutral agents [41].

Till proved otherwise, alogliptin is safe and effective in treating type 2 diabetes as add-on or monotherapy. The number of patients who discontinued their medication due to adverse events while receiving either doses of alogliptin was not different from the number patients who were receiving placebo or other antidiabetic drugs. Similarly, alogliptin treated patients with type 2 diabetes did not experience alogliptin related adverse events and there were no significant changes in serum lipids level. However, the authors of this meta-analysis share the concerns of other investigators on the possible adverse events of incretin-based therapies, such as pancreatitis, anaphylaxis and Steven Johnson’s syndrome [42-45], which were not assessed in this meta-analysis due to lack of data.

As limitations, this meta-analysis has noted a high degree of heterogeneity among the included studies. The possible explanation for the inconsistencies across studies could be: the variation in the duration of therapies, the difference in the sites of the studies, the type of antidiabetic drugs used with alogliptin and for controls, patients’ difference in their antidiabetic drug experience and the type of anidiabetic drugs used before the start of alogliptin therapy. Secondly, all the included studies were sponsored by a pharmaceutical company, in which the findings might ave been manipulated as noted by other authors [46,47]. Thirdly, since the included studies for meta-analyses of adverse events and change in serum lipids were few and these studies were not primarily designed to assess adverse events and lipid profile change, the findings might not be conclusive. This is because; sample sizes of the individual studies included in a meta-analysis did not provide adequate power to test rare adverse events [48]. Fourthly, this meta-analysis was not able to incorporate studies written in other languages.

## Conclusion

In conclusion, regardless of the dose and antidiabetic drug treatment history, alogliptin alone or in combination with other antidiabetic drug(s) has significantly reduced HbA1c and FPG in patients with poorly controlled type 2 diabetes. Furthermore, the proportion of patients experiencing an adverse event, discontinuation due to adverse events, and the effect on serum lipids was not different from placebo or other antidiabetic drugs. However, the effect of alogliptin on body weight change and its consistent efficacy and safety for longer duration of therapy needs further investigation.

## Competing interests

The authors declare that they have no competing interests.

## Authors’ contribution

AB conceived the study, conducted the literature search, performed the statistical analyses and participated in the study selection and manuscript writing. YB participated in articles selection, in manuscript writing and assisted in statistical analyses. Both authors read and approved the final manuscript.

## Pre-publication history

The pre-publication history for this paper can be accessed here:

http://www.biomedcentral.com/1472-6823/13/9/prepub
